# From Planning to Implementation of the YouthCan IMPACT Project: a Formative Evaluation

**DOI:** 10.1007/s11414-019-09658-4

**Published:** 2019-07-24

**Authors:** Joanna Henderson, Margaret Hess, Kamna Mehra, Lisa D Hawke

**Affiliations:** 1 Margaret and Wallace McCain Centre for Child, Youth and Family Mental Health, Centre for Addiction and Mental Health, Toronto, ON Canada; 2 Department of Psychiatry, University of Toronto, Toronto, ON Canada

## Abstract

In order to improve the youth mental health system, there is an international movement toward developing community-based service hubs that provide integrated, collaborative care to youth. However, the implementation of multisystem collaboration is complex and can be hampered by barriers. This paper presents a formative evaluation of the YouthCan IMPACT integrated youth services project based on the Consolidated Framework for Implementation Research (CFIR), to identify facilitators and barriers to successful implementation. Results highlight that previous positive working relationships along with collaborative investment of resources from partnering organizations are essential to implement an integrated youth service model. In addition, it is important that representative members of all key stakeholder groups, including staff, youth, and caregivers, be involved in the development and execution of the project to ensure effective implementation. Attention to the facilitators and barriers to implementation may help teams seeking to implement highly collaborative, integrated models of service delivery for youth in the community.

## Introduction

Adolescence marks the developmental transition from the dependency of childhood to the independence of adulthood.^1^ During this critical time, an estimated 12.6% of Canadian youth experience mental illness.^2^ Mental illnesses can be chronic and disabling, with wide-reaching effects on quality of life, productivity, and functioning.^3^ Adolescence is an especially important period, since over 70% of mental illnesses first present before adulthood.^4^ Furthermore, suicide is the second leading cause of youth mortality.^5^ Despite the clear need for prevention and early intervention services, youth worldwide face barriers with regard to service acceptability, accessibility, availability, and equity.^6^

In Ontario, Canada, the youth mental health system has been described by youth as lacking in resources and ineffective, with disjointed services.^7^ In addition, many Canadian families seeking services from the child and youth mental health system have found it difficult to identify and navigate available and appropriate services, and they experience long waitlists, as well as a deficiency of access to coordinated care.^8^ Service users’ (e.g., youth and caregivers) involvement in service design and evaluation has been shown to result in better experiences and outcomes, yet their voices are rarely integrated.^9^ Thus, a crisis of care has emerged, which requires innovative solutions.

In order to improve the youth mental health system, diverse agencies are increasingly collaborating to provide youth with a wide range of services based on their needs. Throughout the world, service problems in youth mental health care are being addressed through integrated youth service hubs.^10–13^ These integrated service hubs bring together the expertise of diverse service providers in a “one-stop shop” format, with a focus on increasing access and reducing the siloing of services. These models typically include services for mental health and substance use issues, primary health care, and social and vocational services.^11, 12^ Examples of integrated youth service hub models in Canada include YouthCan IMPACT in Toronto,^12^ Foundry in British Columbia,^14^ ACCESS Open Minds, a pan-Canadian initiative,^15^ and Ontario’s Youth Wellness Hubs Ontario initiative.^16^ Examples in other countries include headspace in Australia,^17^ Jigsaw in Ireland,^18^ and the Youth One Stop Shops in New Zealand.^19^ California and Quebec both recently announced plans to develop such models.^20, 21^

**Figure 1:**
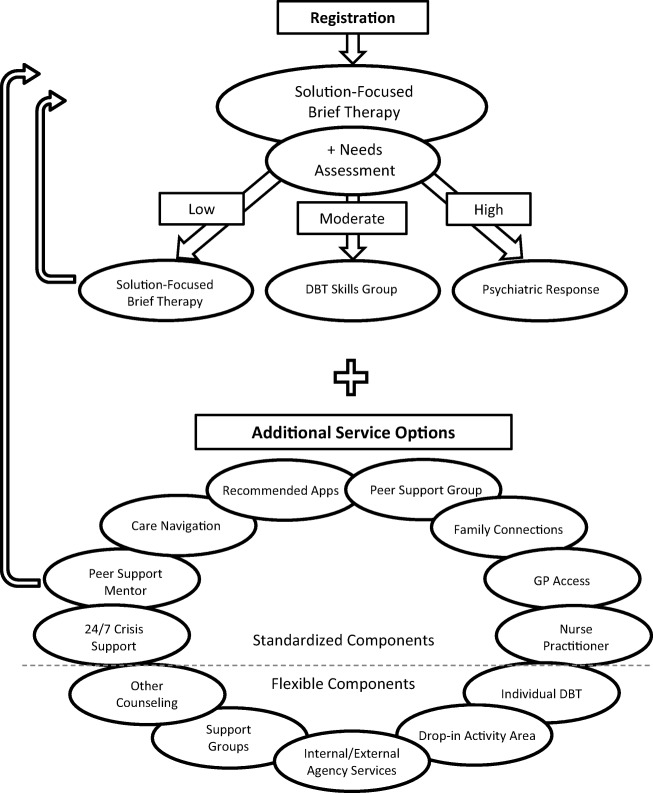
YouthCan IMPACT service delivery model

The YouthCan IMPACT initiative in Toronto, Canada, includes an integrated youth services model with an embedded pragmatic randomized control trial (pRCT).^12^ The pRCT compares the YouthCan IMPACT integrated youth services (YCI-IYS) model (Fig. 1) to out-patient hospital-based psychiatric services, to determine the impact on clinical and functional outcomes, user satisfaction, and cost-effectiveness. The YCI-IYS model is community-based and was co-created with youth,^22^ family members, and other stakeholders. It aims to provide accessible, youth-friendly,^23^ evidence-informed mental health services, tailored to the needs of individual youth and offered in the context of other health and social services.

The YouthCan IMPACT integrated youth services model^12^ is unique in its design and co-creation with various stakeholders to establish youth- and family-friendly services that are relevant to youth and better able to meet their needs. Based on feedback from stakeholders, the stepped-care model includes a brief standardized needs assessment, solution-focused brief therapy (SFBT),^24, 25^ dialectic behavioral therapy (DBT) skills groups,^26^ psychiatric services, and other diverse services available on site, such as nurse practitioner services, care navigation, individual and group peer support, e-resources, and interventions for caregivers, including the Family Connections DBT-based caregiver group.^27^ The YCI-IYS model is consistent with the movement toward integrated youth service hubs; however, it is unique in that it was co-created by local service providers, youth, caregivers, and researchers with a view to creating an accessible, youth- and family-friendly suite of services that leverages available services to be locally feasible, while also being highly relevant to youth and caregivers, and therefore better able to meet their needs.

Achieving the expected outcomes from the implementation of health service interventions can be significantly hampered by challenges faced in real-world settings.^28, 29^ Thus, conducting a formative evaluation during early project phases can be critical to assessing the potential for the future real-world success of a novel service model. A major benefit of a formative evaluation is that it does not examine traditional summative endpoint measures; rather, it pinpoints potential or actual facilitators and barriers to implementation efforts.^30^ Thus, a formative evaluation can provide important information for achieving replicability and successful implementation of novel service models, like the YCI-IYS model.

The Consolidated Framework for Implementation Research (CFIR)^28^, which has previously been used to guide formative evaluations^31, 32,^ proposes five main domains relevant to implementation success—intervention characteristics, outer settings, inner settings, characteristics of individual, and process—each further divided into constructs influencing implementation.^28^ The CFIR has been effectively used to develop interview questions and frame data collection and data analysis for implementation evaluations.^33^ Furthermore, it has also been used to guide and examine initiatives in community-based youth mental health.^34^ Accordingly, the CFIR framework was used in this study to conduct a formative evaluation of the YouthCan IMPACT initiative, including development and implementation of the YCI-IYS model and the related pRCT.

### Objectives

The main objectives in this study were (1) to describe and examine the YouthCan IMPACT initiative startup process and (2) to determine the facilitators and barriers to implementation and ongoing operations.

Consistent with implementation science recommendations and the call for formative assessments to inform implementation,^30^ it was expected that this evaluation would enhance the implementation of the YouthCan IMPACT initiative and produce knowledge about the implementation process that could be used to optimize the potential launch of additional local YCI-IYS sites. Furthermore, the knowledge generated could be used to assist other groups in implementing similar hub-based models or other youth mental health interventions.

## Methods

### Interviews

Data were collected through semi-structured interviews. Twenty-six members of the YouthCan IMPACT team were interviewed for this evaluation. Participants had diverse project roles, spanning from scientists and partnering agency leads to youth and family representatives and direct clinical service staff. Ethical approval to conduct the study was obtained from the Centre for Addiction and Mental Health Research Ethics Board. All participants were informed about the process and written consent was obtained. Interviews were conducted by research staff at a time and place convenient for the participants. Where it was not possible to conduct interviews face-to-face, they were conducted via telephone. Questions for the interviews were developed based on (a) CFIR domains and (b) information collected from a review of protocol documents, archived meeting minutes, and committee meetings/site visits. Question sets were adapted to the role of each interviewee. The information gathered from the interviews was analyzed and categorized based on the CFIR domains and constructs.^28^

### Surveys

In addition to semi-structured interviews collected from YouthCan IMPACT team members, 26 direct service staff and managers at the clinical sites implementing the YCI-IYS model were surveyed at the beginning of implementation. Ethical approval for the surveys was also obtained from the Centre for Addiction and Mental Health Research Ethics Board. The participants were approached in person, consent forms were administered, and participants completed a survey. The survey questions included demographic information and the Service Provider Adopter and Innovation Characteristics Questionnaire (SPAICQ).^35, 36^ The SPAICQ was created by adapting previously used measures^35–39^ and formulating new items based on the theoretical constructs from the literature. The SPAICQ has three subscales examining adopter characteristics (concern, self-efficacy, and attitudes) with a total of 21 items, as well as three subscales examining innovation characteristics (complexity, compatibility, and relative advantage), with a total of 14 items. Response options are on a 7 point Likert scale ranging from 1 “strongly disagree” to 7 “strongly agree.” Table 1 provides sample items in each subscale. Demonstrating reliability, the internal consistency coefficients for each subscale (Cronbach's alpha) were found to range from 0.76 to 0.87 (Table 1). The data obtained from the survey were analyzed using SPSS 24.

**Table 1:** Service Provider Adopter and Innovation Characteristics Questionnaire

Variable subgroup component	Sample item	No. of items	Cronbach’s alpha	Mean	S.D
Adopter characteristics					
Concern	I believe it is important to provide services that are more integrative and collaborative than current hospital-based services.	5	0.83	6.57	0.71
Self-efficacy	I can intervene effectively to address the co-occurring mental health and substance use needs of youth in an integrated, collaborative way.	4	0.85	5.16	1.47
Attitudes	Addressing integrative, collaborative care concerns improves the quality of my agency	12	0.87	5.49	0.86
Innovation characteristics					
Complexity	Addressing the mental health and substance use needs of youth in an integrated, collaborative way is difficult to do.	5	0.76	4.18	1.17
Compatibility	Addressing the mental health and substance use needs of youth in an integrated collaborative way fits in well with my organization.	4	0.79	6.03	0.85
Relative advantage	In general, addressing the mental health and substance use needs of youth in an integrated, collaborative way is more effective in creating attitudes that support recovery than practices focused on one area of concern or one type of intervention.	5	0.85	6.15	0.83

## Results

Results describe the qualitative findings, with quantitative findings embedded to facilitate triangulation. Complete quantitative findings are summarized in Table 1.

### Description of the startup process

**Figure 2:**
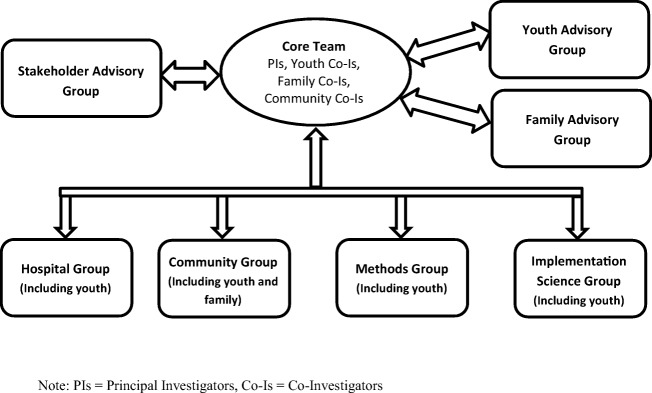
YouthCan IMPACT governance model

A number of researchers, service providers, clinicians, youth, and family members came together through their strong interest in youth mental health, their complementary expertise, and prior working relationships to develop an integrated youth services project in response to a call for grant proposals. Two youth with lived experience participated as co-investigators and co-creators of the initiative and a broader range of youth were engaged in project development through a pre-existing National Youth Action Council (NYAC).^40^ Similarly, one caregiver was engaged as a co-investigator on the project and a Family Advisory Group was established to provide a broader range of caregiver feedback. Service partners included child and youth mental health agency representatives, youth social service providers, primary care providers, and hospital-based youth psychiatry clinicians. Other stakeholders who could inform the methodology, design, economic analysis, and policy context were also engaged for the project. After funding was awarded by the Ontario SPOR Support Unit, research staff and additional youth were hired as YouthCan IMPACT staff to support implementation of the project and a local Youth Advisory Group was established. In addition, sub-project working groups were developed to ensure the necessary leadership and accountabilities were in place to achieve the project deliverables. To ensure effective collaboration and communication between team members, a governance model was created (Fig. 2).

In order to develop the YCI-IYS model and select the interventions that would be offered as part of the model, community partners, youth, and caregivers worked together with the researchers to develop a pathway of services incorporating sustainable interventions already working effectively at some or all of the community agencies. These interventions were then organized into a comprehensive stepped-care YCI-IYS service delivery model (Fig. 1). In addition, the roles and responsibilities of partnering agencies were outlined, and memorandums of understanding, contracts, and budgets were established. As well, an intervention manual that was created for training on-site staff about the model and fidelity measures were put in place to support consistent implementation of the model. An incremental rolling start approach was taken, where agencies possessing the capacity started implementing the model while others continued to develop and become ready to implement. Once workflow processes were established, services were promoted in the community and expanded. Fidelity check points were also implemented to permit ongoing implementation monitoring and adjustment.

### Barriers and facilitators of implementation

To develop an in-depth understanding of the barriers and facilitators of implementation of the YouthCan IMPACT initiative, we used the CFIR framework.^28^ The barriers and facilitators were identified via both interviews and questionnaires, and are presented based on the five domains within the framework provided by CFIR.^28^

### Domain 1: intervention characteristics

According to interviewees, the YCI-IYS model was primarily designed by partnering agencies, youth, and caregivers with support from the project leads and project coordinator. Interventions proposed by youth, caregivers, and community agencies were included after ensuring evidence-based support. Thus, it was perceived as a “bottom-up” community-based model rather than a “top-down” imposed research initiative. This was identified as an important aspect of the model for community service-provider uptake, and service providers who were not involved in model creation were informed of this aspect of the development process to facilitate uptake.

Key constructs of an intervention considered as part of CFIR include the strength of evidence supporting the intervention and its perceived relative advantage over interventions in use.^28^ In this project, interviewees reported that partner buy-in to the model was enhanced by evidence of international success of similar hub-like models,^41, 42^ along with the evidence base for the individual interventions included in the pathway.^25–27, 43^ In addition, partnering organizations had a history of using these interventions successfully. Many team members reported that they had been frustrated with the youth mental health system at the time and that they were highly motivated to provide better services for youth. These findings are reflected in the quantitative data; surveys of hub staff and managers revealed that service providers perceived the YCI-IYS model to have high relative advantage over existing youth mental health services (relative advantage subscale mean score = 6.15, S.D. = 0.83; see Table 1). Thus, qualitative and quantitative data converge to suggest that the implementation of the model was facilitated by strong evidence-based support and a high perceived relative advantage over the existing youth mental health system.

Adaptability of the model, which is another key CFIR feature, was identified as a strength by interviewees, who indicated that the model was broken down into core components (SFBT, DBT, and psychiatry) and adaptable components (other services available at each site). They also reported, however, that a challenge that emerged during the initial implementation phase was balancing adaptability with fidelity. In order to facilitate fidelity, the team aimed to partner with organizations that already provided the core services. When an agency was not able to provide the core services, other partnering organizations leveraged their existing service capacity to provide the missing services for these agencies or training in these services. By leveraging the strengths of each partner agency in the core components, interviewees reported it was possible to enhance fidelity while maintaining adaptability. Overall, according to interviewees, the adaptability of the model was considered a facilitator of these early phases, as it ultimately allowed for the implementation of the model across three different sites with variable local demands, different combinations of services provided, and different existing capacities (e.g., financial resources, staff size, and existing interventions).

The complexity of an intervention can be a barrier to its effective implementation.^28^ Qualitatively, interviewees identified that the YCI-IYS model is streamlined, with only one step to access walk-in services and a further step to access needs-based services. Thus, the model itself is not complex. The most complex aspect of the YCI-IYS model, as expressed by the team members interviewed, is the collaboration among multiple organizations with different intervention processes at each organization. This was also reflected in the quantitative data, where the service providers felt the model was neither highly complex nor highly straightforward to implement (complexity subscale mean score = 4.14, S.D. = 1.16; Table 1). Interviewees qualified this finding by indicating that the relative complexity of the intervention was reduced by leveraging the relationships with multiple organizations, which helped to provide inexperienced staff with experience by allowing them to “shadow” experienced staff in other organizations.

Another challenge identified as present during the implementation phase was the cost of the intervention, since the services that could be feasibly offered were limited due to the budgetary restrictions. Again, leveraging existing services helped address this barrier. Despite this challenge, interviewees indicated that staff still highly regarded the design of the model and felt that it was not compromised by the budget.

### Domain 2: outer settings

Interviews revealed that the YouthCan IMPACT project was created during a time of movement toward community-based mental health services for youth in Ontario and across Canada, reflecting the CFIR domain “outer setting.” Strategic planning by the provincial government at the time^44^ aimed to ensure children, youth, and families had accessible responsive local community-based mental health services. Lead agencies responsible for the provision of core youth mental health services in their communities had recently been identified. Participants felt that the YCI-IYS model, which was also aiming to strengthen community-based mental health services to make them more accessible for youth, was well aligned with this movement. In addition to the provincial government plan,^44^ there was a national movement to conduct “patient-oriented” research (Strategy for Patient-Oriented Research (SPOR)^45^) with both provincial and federal funding streams as well as cross-jurisdictional interest in health systems integration. Interview participants highlighted that YouthCan IMPACT goals aligned with these aspects of the external environment, facilitating partner participation and uptake of the model.

According to interviewees, development and implementation of YouthCan IMPACT was facilitated by the high degree of social capital and strong ties with local community mental health organizations that the project leads had previously established. They also had ties within their affiliated hospitals and multiple domains of academia. Furthermore, members of the YouthCan IMPACT project had boundary-spanning roles and broader experience due to engagement with multiple organizations. Since increased social capital and relationships with individuals who possess boundary-spanning roles have been known to increase the efficiency of implementation,^46, 47^ the current project appeared to have the benefit of robust outer settings facilitators.

### Domain 3: inner settings

The way in which an organization is structured can greatly impact the implementation success of that organization.^48^ In order to facilitate the necessary formation of a cohesive group to implement a project as highly collaborative as the YouthCan IMPACT project, interviewees highlighted the importance of the governance model, with a core team of the five project leads together with other team members, as well as the subdivided, specialized working groups (Fig. 2), in providing structure to the inner setting. Each working group included a project lead and the project coordinator. According to interviewees, this helped to create decentralized decision-making opportunities, as well as to ensure effective communication across the working groups and to the core team. In addition, interviewees indicated that the project co-ordinator served as a champion for the project outside of the governance structure,^49, 50^ facilitating and communicating effectively across the partner organizations.

Relationships play an important role in implementation^51^ and this notion was strongly supported by the YouthCan IMPACT team. Interviewees reported that positive previous working relationships were a key factor in the determination and persistence of the partnering organizations. They indicated that this aspect of the inner setting was a key facilitator of startup and implementation, as they felt they could trust their teammates, voice their ideas, and disagree openly, while they felt confident that the sharing of resources would result in mutual benefit. Beyond the relationships among project leads and community partners, positive working relationships were found to be critical to meaningfully engage youth and families. There is a power differential between service users and their health services providers, making service users vulnerable.^52^ Since the project leads were experienced in maintaining effective relationships with youth and caregivers,^40^ youth and adult partners alike felt the respectful and open atmosphere during the decision-making process in this project allowed all parties to contribute equally and meaningfully.

Interviews also revealed that leadership engagement in the YouthCan IMPACT project was a key facilitator and one of the main drivers of the startup and implementation processes. Key leaders within partnering organizations, who had decision-making power on behalf of the organizations, were engaged, which allowed the development process to move efficiently. Through surveys of hub staff and managers, quantitative findings revealed that the YCI-IYS model was felt to be highly compatible with the work of their organizations (compatibility subscale mean score = 6.03, S.D. = 0.85). Interviewees indicated that the project was given a high degree of priority from organizational leaders and staff; although the project required frequent meetings and a substantial time commitment, resulting in possible short-term reductions in productivity, organizational leaders had the foresight to provide this time on an in-kind basis for the resulting long-term gain. This endurance, coined “managerial patience,” has been found to result in implementation success.^53^

One of the challenges reported by interviewees in the YouthCan IMPACT project was a lack of control over the architecture of the partnering organizations. Research has shown that stable teams are more likely to implement a project successfully.^51^ This was observed in the current project, where organizations with consistent management throughout the startup phase reported finding it easier to implement the project than partners with fluctuating management resources; organizational mergers occurring during the startup phase posed challenges in terms of structural stability. In addition, interviewees indicated that communication within partnering organizations, i.e., between higher level management and direct service staff, was challenged by decisional changes over time during the initial project phases. It was found to be helpful to include direct service staff in the decision-making process and/or provide finalized information to direct service staff in order to facilitate implementation. Interviewees observed that there was a shift in the YCI-IYS model implementation sub-project working group as it moved from upper-management community representatives during planning and design to operational, clinical managers during the implementation phase.

### Domain 4: characteristics of individuals

Individuals must have sufficient information about an intervention to be willing to adopt it.^46^ While the members of the YouthCan IMPACT core team and working groups involved in the design of the model had “expert” knowledge of the model, the direct service staff, despite the startup training provided, expressed that they would have benefitted from more information about the project during the startup phase. These same staff, however also held attitudes typically found to facilitate adoption and implementation. For example, the quantitative data reveal that respondents perceived taking an integrated approach to mental health care to be important (concern subscale mean score = 6.57, S.D. = 0.71) and held positive attitudes about integrated collaborative care (attitudes subscale mean score = 5.49, S.D. = 0.86).

Self-efficacy positively affects individual’s willingness to adopt an intervention, their persistence in face of difficulty, and their performance.^54^ In the current project, quantitative data showed moderately high self-efficacy among staff to deliver the selected interventions (self-efficacy subscale mean score = 5.16, S.D. = 1.47). In order to further improve self-efficacy, staff were provided time and experience by shadowing other experienced staff. In addition, qualitative data revealed that that organizational managers realized the importance of hiring candidates experienced in providing services included in the project. For example, partner organizations specifically hired staff with experience offering brief walk-in services, a pillar of the YCI-IYS model. Interviewees reported that individuals with prior experience with these services expressed greater confidence in their ability to offer the YouthCan IMPACT services.

One important facilitator during early phases identified by interviewees was the individual traits of team members involved in the YouthCan IMPACT project. Interviewees indicated that during the development phase, the individual team members had a “yes” mentality, i.e., when encountering issues during implementation, rather than starting the discussion with “no,” they tried to develop solutions based on available resources. For example, if organizations felt unable to support a core component of the model, the team members offered creative solutions to provide the needed support. Interviewees highlighted the strength of individual characteristics as facilitating implementation of the model, as the individuals formed a highly determined group with a desire to be successful and persistence to accomplish the team’s goals.

### Domain 5: process

According to the interviewees, during the planning process of the YouthCan IMPACT project, the main focus was on enabling and empowering community partners for implementation. Some examples provided include the team’s efforts to ensure that the community partners guided the selection of interventions in the YCI-IYS model, preparing staff members to implement the model by providing them with training and conducting an incremental implementation, allowing time to build the capacity of the organizations to provide the services included in the project. Interviewees reported that these processes were key: the quality and extent of planning for implementation during the startup phase facilitated the implementation through clear and feasible design, staff preparation, and incremental execution.

Some interviewees noted that a facilitator to building this group of team members was the project leads’ experience and dedication to engaging youth and caregivers in mental health research and program development.^40, 55^ This helped the rest of the YouthCan IMPACT team to support the youth and caregiver’s ongoing involvement since they were guided by experienced leadership. Interviewees reported that another major facilitator to youth and caregiver engagement was the work done by the youth and caregiver co-creators themselves to engage and maintain a large pool of involved individuals in the Youth Advisory and the Family Advisory Groups, respectively.

A key construct of the implementation process is reflecting on and evaluating the progress of the implementation.^28^ Interviewees highlighted the fact that the YouthCan IMPACT team conducted regular debriefing meetings with clinical hub managers and the project coordinator, which allowed for the identification and tackling of implementation challenges. Such challenges included clarifying the needs-based care model and updating the implementation plans that evolved during the startup process to ensure fidelity of all core components of the YCI-IYS model. Interviewees indicated that with efficient communication, the fidelity of the finalized model among direct service providers was supported, which led to uptake of the final model.

## Discussion

The main objectives of this study were to describe the startup process of the YouthCan IMPACT project and examine the barriers and facilitators encountered during this process. Using surveys and interviews, information was gathered and analyzed based on Damschroder’s CFIR constructs for implementation research.^28^ The results provide specific findings applicable to the ongoing optimization of the YouthCan IMPACT project in particular, but also general findings that other integrated youth service hub stakeholders can consider to optimize their implementation and development processes.

The startup process involved the formation of a team of project leads, youth, caregivers, community mental health and hospital clinicians and decisionmakers, researchers, the project coordinator, and other stakeholders to co-create and implement the YouthCan IMPACT initiative. Key facilitators during the early startup and implementation phases examined in this study included the development of the YCI-IYS model using a “bottom-up” co-design approach with built-in adaptability as well as its capacity to address an important issue with an evidence-supported approach that was perceived to have advantages over existing practices, while at the same time not being perceived as too complex. In addition, the initiative was happening in a broader context of positive provincial and national momentum regarding integrated youth services and the team involved key members that had boundary-spanning roles. Within the partner organizations and the YouthCan IMPACT team, the governance model with decentralized decision-making, strong individual self-efficacy, positive problem-solving attitudes (“yes” approach), “managerial patience,” expertise in youth and family engagement, high social capital, and the positive previous working relationships among stakeholders facilitated implementation success. Lastly, collaboration and team processes that were perceived as enabling and empowering, as well as comprehensiveness in implementation planning, allowed the model to be implemented in ways that fit with existing capacities. Reflection and adjustment throughout also facilitated engagement and ultimately implementation.

Key barriers to implementation included structural instability within some partner organizations, budgetary challenges, achieving fidelity across sites, and the initial limited integration of direct service staff in the development of the model. These barriers were overcome by effective leadership, relationship trust that allowed for honest discussion, and the involvement of operational managers in the implementation process. In sum, important factors captured by the CFIR framework supported the successful implementation of the YouthCan IMPACT initiative, including aspects of the inner and outer setting, intervention characteristics, individual characteristics, and process.^28^

There is increasing provincial, national, and international interest in developing and implementing integrated youth service hubs to address systems challenges. Integrated youth service like the YouthCan IMPACT model provides a wide range of coordinated services to youth (e.g., mental health services, physical health services, social services etc.) in a “one-stop shop” format.^17^ Similar models have been developed across Canada and around the world.^14–19^ Research has been conducted regarding various aspects of these service hubs, including youth profiles, service use, and outcomes.^11, 12^ Ongoing evaluation of additional aspects, including the implementation process, is critical to continuing to optimize processes in this growing systems transformation movement. CFIR has been used to conduct formative evaluations of diverse health service implementation initiatives.^56–58^ However, to the best of our knowledge, the startup process and the barriers and facilitators involved in the development of integrated youth service hubs have not been documented using the CFIR framework.

An important question faced by the stakeholders in the integrated youth service hub landscape is the sustainability of these hub models. Each model has its own funding structure, including government health funding and philanthropic funding, in some cases supplemented by research funding. The YCI-IYS model aims to leverage existing services whenever possible. By co-designing the model with key stakeholders to address widely recognized system and service gaps affecting these stakeholders, and by restructuring existing services through creative partnerships to optimize service delivery rather than building new services, sustainability was enhanced from the outset. As research findings continue to emerge supporting the effectiveness of such models, it will be important that policymakers recognize the benefits of integrated youth service hub models and ensure that the funds are available to support them on an ongoing basis.

Based on our findings from the startup phase of the YouthCan IMPACT project, we suggest that it is feasible and effective to tackle the deficits in the current mental health system with innovative and collaborative efforts. This project confronts the deficits in youth mental health system by collaborating with existing services rather than replacing them by a new set of services.^12^ Although implementation of a highly collaborative project may face barriers,^59–62^ these barriers can be overcome through strong leadership and stakeholder involvement throughout the project phases, thus optimizing the implementation process. The current findings go hand-in-hand with a YouthCan IMPACT Implementation Guidebook,^63^ which aims to support teams interested in implementing community-based integrated service hub models of youth mental health service delivery.

### Limitations

Limitations of this study include the possibility that social desirability could have affected participants’ responses during interviews and surveys. This was mitigated by having an unfamiliar research staff member conduct the interviews and review surveys for identity-revealing information and anonymize these responses. In addition, this study describes the startup process of an integrated youth services model with heavy research involvement and in an urban setting. The same process may not be applicable to developing hub-like models in other contexts (non-research) or in other settings (e.g., rural settings). Research results on the effectiveness of the model are not yet available, although work is in progress.^12^

## Implications for Behavioral Health

Understanding the process of implementing an integrated youth service hub is crucial, since the system is moving toward this type of holistic approach for youth mental health and substance use concerns.^11–13^ This formative evaluation explores the potential barriers and facilitators of successful implementation of an integrated youth services model of service delivery. It can be used by existing, as well as future, hubs to understand potential barriers and facilitators to implementation, including the importance of positive partnerships and persistence. Involvement of service users beginning during early development and through implementation helps not only in engaging them, but also to ensure services that are designed to address their needs. Evaluations, such as this formative evaluation of YouthCan IMPACT’s start-up and early implementation phases, are essential for enhancing implementation of services for youth with mental health and addictions concerns.
